# Actual incidence of cerebral infarction after thoracic endovascular aortic repair: a magnetic resonance imaging study

**DOI:** 10.1093/icvts/ivab240

**Published:** 2021-10-11

**Authors:** Sohsyu Kotani, Yoshito Inoue, Naohiko Oki, Hideki Yashiro, Takashi Hachiya

**Affiliations:** Department of Cardiovascular Surgery, Tokai University School of Medicine, Kanagawa, Japan; Department of Cardiovascular Surgery, Tokyo Dental College Ichikawa General Hospital, Chiba, Japan; Department of Cardiovascular Surgery, Hiratsuka City Hospital, Kanagawa, Japan; Department of Radiology, Hiratsuka City Hospital, Kanagawa, Japan; Department of Cardiovascular Surgery, Saiseikai Yokohamashi Tobu Hospital, Kanagawa, Japan

**Keywords:** Cerebral infarction, Thoracic endovascular aortic repair, Diffusion-weighted magnetic resonance imaging

## Abstract

**OBJECTIVES:**

The actual incidence of cerebral infarction (CI), including asymptomatic infarction, owing to thoracic endovascular aortic repair (TEVAR) has not been reported in detail. This study was performed to investigate the incidence of post-TEVAR CI by using diffusion-weighted magnetic resonance imaging (DW-MRI) and to determine the risk factors for both symptomatic and asymptomatic CI.

**METHODS:**

We examined 64 patients undergoing TEVAR at our institute between April 2017 and November 2020. Aortic atheroma was graded from 1 to 5 by preoperative computed tomography. Cerebral DW-MRIs were conducted 2 days after the procedure to diagnose postoperative CI.

**RESULTS:**

A total of 44 new foci were detected by post-interventional cerebral DW-MRI in 22 patients (34.4%). Only one patient developed a symptomatic stroke (1.6%), and TEVAR was successfully completed in all cases. Debranching of the aortic arch and left subclavian artery occlusion with a vascular plug was performed in 19 (29.7%) and 12 (18.8%) patients, respectively. The number of patients with proximal landing zones 0–2 was significantly higher in the CI group than in the non-CI group (68.2% vs 11.9%; *P* < 0.001). The following risk factors were identified for asymptomatic CI: aortic arch debranching (*P* < 0.001), left subclavian artery occlusion (*P* = 0.001) and grade 4/5 aortic arch atheroma (*P* = 0.048).

**CONCLUSIONS:**

Over one-third of the patients examined by cerebral DW-MRI after TEVAR were diagnosed with CI. High-grade atheroma and TEVAR landing in zone 0–2 were found to be positively associated with asymptomatic CI.

**Clinical trial registration:**

02-014.

**Figure:**
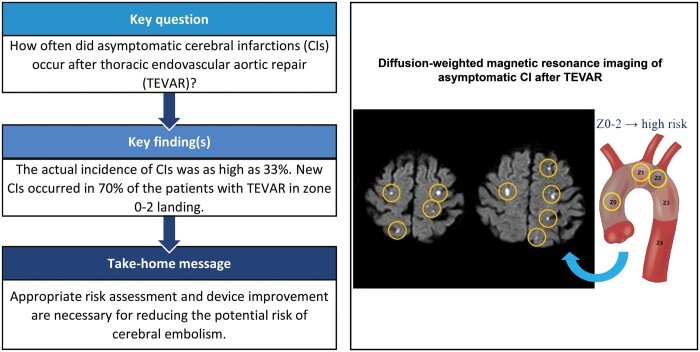

## INTRODUCTION

Cerebral infarction (CI), which remains one of the major complications after thoracic endovascular aortic repair (TEVAR), has been reported by a large meta-analysis to occur in 4.1% of patients undergoing TEVAR [1].

Conversely, previous studies have shown that asymptomatic CI occurs much more frequently after different catheter-based procedures [2–5]. However, there is a dearth of research on the incidence of asymptomatic CI after TEVAR. Asymptomatic CI is any image-proven ischaemic brain injury in patients with no focal neurological abnormality. These lesions have been identified as independent predictors of future stroke, dementia, depression and cognitive impairment [6, 7]. The risk of asymptomatic cerebral embolism after TEVAR is yet unknown. Here, we used preoperative and postoperative serial diffusion-weighted magnetic resonance imaging (DW-MRI) to evaluate the actual incidence of CI, including asymptomatic CI as well, in patients undergoing TEVAR, and to evaluate the risk factors for asymptomatic CI.

## PATIENTS AND METHODS

### Ethical statement

This study was approved by the institutional review board of Hiratsuka City Hospital (7 April 2017, reference number: 02-014). The requirement of a written, informed consent was waived by the institutional review board.

### Patient population

A total of 69 patients treated with TEVAR as an elective procedure for both degenerative aneurysms and chronic dissections at our institute between April 2017 and November 2020 were analysed. Patients who underwent TEVAR for residual aneurysms or dissections after total arch replacement (with both classical elephant trunk and frozen elephant trunk) were also included. Patients undergoing emergent or urgent procedures, known to have mental disorders, or contraindicated for MRI due to various reasons (e.g. pacemaker and claustrophobia) were excluded. Finally, 64 consecutive patients were included in this study. If the patient complained of symptoms of weakness or numbness or if neurological deficits were detected, neurologists were consulted and a full neurological examination was performed. Symptomatic stroke was defined as focal or global neurological deficits persisting for >24 h with radiographic abnormalities, confirmed by imaging studies using MRI and computed tomography. A patient with clinical infarction initiated antiplatelet therapy (aspirin 100 mg) after TEVAR. Other cases with asymptomatic infarctions did not use of anticoagulants/antiplatelets because the cause of infarctions was embolus, not thrombus. Unless a history of thromboembolism such as CI, atrial fibrillation or post-coronary artery bypass grafting before TEVAR, the patients did not receive anticoagulant/antiplatelet therapy. Ethical approval was obtained from the Ethics Committee of Hiratsuka City Hospital, which waived the need for informed consent.

### Surgical procedure

**Figure 1:**
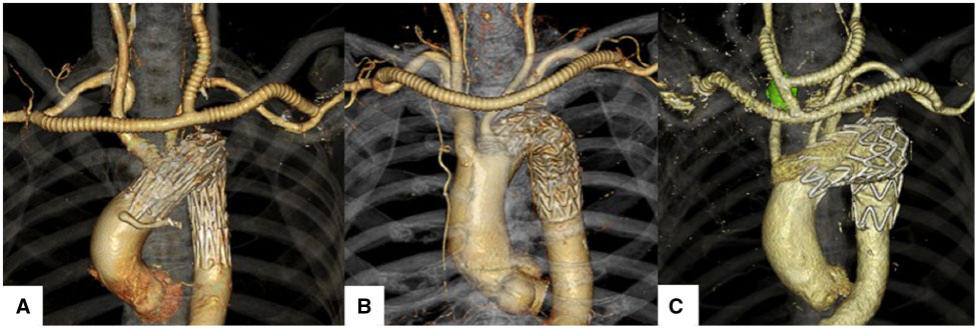
(**A**) Zone 0 landing thoracic endovascular aortic repair using Najuta semicustom-made fenestrated stent graft with right axillary artery (AxA)-left common carotid artery-left AxA bypass. (**B**) Zone 2 landing thoracic endovascular aortic repair with right AxA-left AxA bypass. (**C**) Zone 1 landing thoracic endovascular aortic repair for arch aneurysm with right aberrant subclavian artery.

Prior to the TEVAR procedure, surgical revascularization was performed to obtain a sufficient length of the proximal landing zone for stent-graft implantation. For TEVAR landing in zone 0, we used a fenestrated stent graft following an extra-anatomical bypass from the right axillary artery (AxA) to the left AxA and the left common carotid artery (CCA) using an 8 mm, ringed, expanded polytetrafluoroethylene graft (W. L. Gore & Associates, Flagstaff, AZ, USA). For TEVAR landing in zone 1, we used a tubular stent graft following a bypass from the right AxA to the left AxA and the left CCA (Fig. 1A). For TEVAR landing in zone 2, we conducted a bypass from the right AxA to the left AxA (Fig. 1B). One patient with a distal arch aneurysm and an aberrant right subclavian artery (SCA) received a right CCA-right AxA-left CCA-left AxA bypass (Fig. 1C). Amplatzer vascular plugs (AGA Medical Corp, Plymouth, MN, USA) were used to prevent type II endoleaks at the end of the TEVAR in patients with aneurysms directly involving the left SCA or aneurysms with wide necks. TEVAR, cervical debranching and left SCA plugging were performed as a single-stage procedure under general anaesthesia in a hybrid operating room.

The implanted stent grafts included Valiant Captivia (*n*  =  40; Medtronic, Minneapolis, MN, USA), cTAG (*n*  = 12; W. L. Gore & Associates, Flagstaff, AZ, USA), Zenith TX2 (*n*  = 6; COOK MEDICAL LLC, Bloomington, IN, USA), Relay Plus (*n*  =  3; Bolton Medical, Sunrise, FL, USA) and Najuta (*n*  = 3; Kawasumi Labo. Inc., Tokyo, Japan), with diameters of 26–40 mm and lengths of 90–223 mm (Table 1). To avoid air embolism, catheters and stent-graft delivery systems were carefully flushed with saline. Heparin was administered intraoperatively to maintain the activated clotting time above 200 s. All procedures were performed via standard endovascular techniques. The femoral artery was dissected and used as an access route in all patients. Pharmacologically induced hypotension was performed for TEVAR with proximal landing in zone 0–2. Arterial pressure was monitored with an intra-arterial catheter, and the mean arterial pressure was maintained above 80 mmHg after stent-graft deployment.

**Table 1:** Preoperative and procedural characteristics

	Overall	CI	No-CI	*P*-value
	*N* = 64	*N* = 22	*N* = 42	
Age	73.9 (SD: 9.6)	74.1 (SD: 10.6)	73.7 (SD: 9.1)	
Sex (female)	15 (23.4)	3 (13.6)	12 (28.6)	0.18
Pathology				0.62
Aneurysm	41 (64.1)	15 (68.2)	26 (61.9)	
Dissection	23 (35.9)	7 (31.8)	16 (38.1)	
After TAR	18 (28.1)	1 (4.5)	17 (40.5)	
Hypertension	62 (96.9)	22 (100)	39 (92.9)	0.20
Hyperlipidaemia	31 (48.4)	8 (36.3)	21 (50.0)	0.30
Diabetes mellitus	15 (23.4)	8 (36.3)	9 (21.4)	0.20
Hyperuricaemia	20 (31.3)	10 (45.5)	12 (28.6)	0.18
Smoking	42 (65.6)	13 (59.1)	25 (59.5)	0.97
Chronic kidney disease	8 (12.5)	5 (22.7)	4 (9.5)	0.15
AAA	8 (12.5)	3 (13.6)	4 (9.5)	0.62
Cardiovascular disease	3 (4.7)	1 (4.5)	2 (4.8)	0.97
Prior CI	21 (32.8)	6 (27.3)	16 (38.1)	0.39
AF	6 (9.4)	1 (4.5)	4 (9.5)	0.48
Atheroma grade	2.88	3.11	2.63	
Grade ≤3		16 (72.7)	40 (95.2)	
Grade ≥4		6 (27.3)	2 (4.8)	0.048
Stent-graft system				
VALIANT	40 (62.5)	15 (68.2)	25 (59.5)	
TAG	12 (18.8)	2 (9.1)	10 (23.8)	
TX2	6 (9.4)	2 (9.1)	4 (9.5)	
Relay plus	3 (4.7)	0 (0)	3 (7.1)	
Najuta	3 (4.7)	3 (13.6)	0 (0)	
Proximal landing zone				
Zone 0–2	20 (31.3)	15 (68.2)	5 (11.9)	<0.001
Zone 3	14 (21.9)	3 (13.6)	11 (26.2)	0.25
Zone 4	30 (46.9)	4 (18.2)	26 (61.9)	<0.001
Operative time (min)	128.8 (SD: 63.4)	181.0 (SD: 79.7)	107.7 (SD: 40.1)	0.013
Blood loss (ml)	70.0 (SD: 168.3)	150.2 (SD: 236.4)	27.9 (SD: 98.2)	0.022
Length of aortic coverage (mm)	192.4 (SD: 94.2)	199.1 (SD: 107.2)	189.2 (SD: 89.0)	0.74
Number of stent grafts	1.44 (SD: 0.50)	1.50 (SD: 0.51)	1.41 (SD: 0.50)	0.57
Right AxA—Left AxA bypass	14 (21.9)	10 (45.5)	4 (9.5)	<0.001
Right AxA—Left AxA- Left CCA bypass^a^	5 (7.8)	5 (22.7)	0 (0)	<0.001
No adjunct procedure	45 (70.3)	7 (31.8)	38 (90.5)	
Left SCA occlusion	12 (18.8)	9 (40.9)	3 (7.1)	0.001

Values are mean (SD) and *n* (%).

a This category included one patient with a distal arch aneurysm and an aberrant right SCA, received a right CCA-right AxA-left CCA-left AxA artery bypass.

AAA: abdominal aortic aneurysm; AF: atrial fibrillation; AxA: axillary artery; CCA: common carotid artery; CI: cerebral infarction; SCA: subclavian artery; TAR: total arch replacement.

### Preoperative evaluation of aortic atheroma

**Figure 2:**
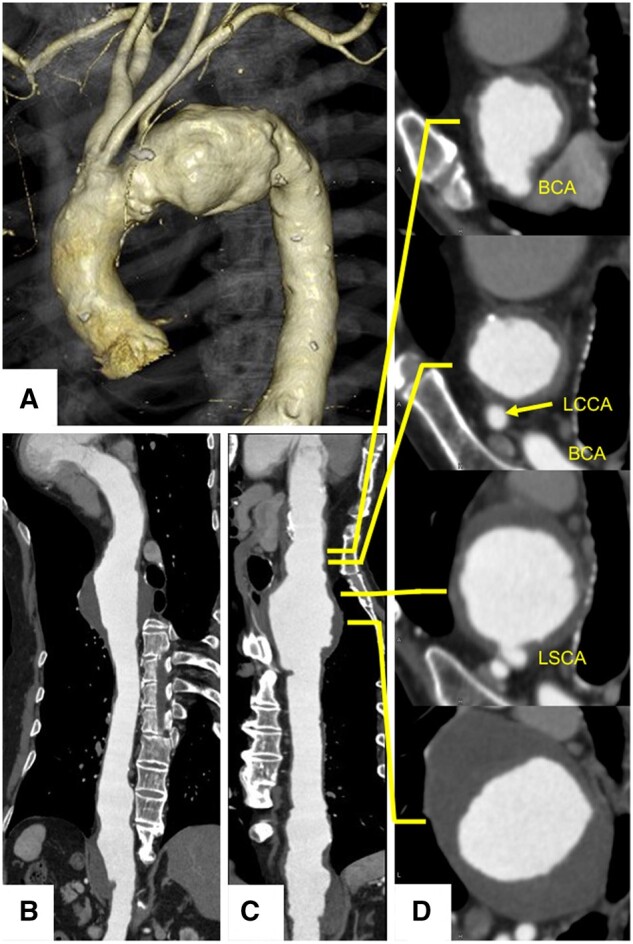
Three-dimensional computed tomography scan (**A**). A centreline image with curved multiplanar reconstruction was created (**B**). The curved multiplanar reconstruction image was converted to a straight multiplanar reconstruction (**C**). The thickness of the atheroma was evaluated from the origin of the brachiocephalic trunk to a section 2-cm distal to the left subclavian artery in the axial image (**D**) linked with the straight multiplanar reconstruction image. This patient is classified as grade 5 with the atheroma 2-cm distal to the left subclavian artery.

All patients underwent preoperative computed tomography angiography (Aquilion ONE, Toshiba Medical Systems, Tochigi, Japan) with intravenous iodinated contrast and a slice thickness of 1 mm. Two independent interventional radiologists examined the aortic arch for the presence of aortic atheroma, calcification and mural thrombus. Any plaque located in the aortic arch within a region extending from the origin of the brachiocephalic trunk to a section 2-cm distal to the left SCA was considered (Fig. 2). After manual analysis of the thickness and composition of the selected plaques, they were graded as previously described by Feezor *et al.* [8]: grade 1, normal; grade 2, intimal thickening; grade 3, atheroma of 5 mm or less; grade 4, atheroma greater than 5 mm; and grade 5, mobile lesion. Proximal landing zones (0–4) were categorized according to the classification proposed by Ishimaru [9].

### Cerebral magnetic resonance imaging

The Discovery MR750w system (GE Healthcare, Little Chalfont, UK) with 3.0-T magnet strength, axial diffusion-weighted imaging and axial T2-weighted fluid-attenuated inversion recovery with 5-mm slice thickness were utilized to perform MRI before surgery (to identify any pre-existing infarct lesions) and 2 days after the procedure. A radiologist blinded to the clinical outcomes of patients read the preoperative and postoperative MRI scans and subsequently reported the number of new lesions. All MRI examinations were assessed twice by the same radiologist.

### Statistical analysis

Continuous variables were presented as mean and standard deviation and categorical variables as frequency and percentage. Depending upon the presence or absence of new lesions on postoperative DW-MRI, we divided the patients into CI and non-CI groups to compare differences in their respective characteristics. Intergroup comparisons were conducted using the Mann–Whitney *U*-test for continuous variables and Fisher’s exact test for categorical variables. A *P*-value of <0.05 was considered statistically significant. To identify the predictors of asymptomatic CI, multivariable logistic regression was performed on variables with a *P*-value of <0.05 in the univariate analyses. Kaplan–Meier analyses were used to compare survival in patients with and without CI and the log-rank test was used to compare differences between survival curves. All statistical analyses were performed using SPSS version 22 software (IBM, Armonk, NY, USA).

## RESULTS

### Patient and procedural characteristics

Table 1 summarizes patient and procedural characteristics. The mean age of the enrolled patients was 73.9 (64.3–83.5) years, and the patient cohort comprised 15 females (23.4%) and 49 males (76.6%). Indications for TEVAR were degenerative aneurysm in 41 patients (64.1%) and chronic type B dissection in 23 (35.9%) cases. We conducted TEVAR for residual aneurysm or dissection after total arch replacement in 18 patients (28.1%), one of whom (5.6%) developed a new cerebral infarct lesion. All patients underwent elective TEVAR and were clinically stable. Twenty patients (31.3%) had TEVAR landing in zone 0–2; 14 (21.9%) in zone 3; and 30 (46.9%) in zone 4. Debranching was required in 19 patients. Of the 4 patients with TEVAR landing in zone 0 or 1, 3 underwent a right AxA-left CCA-left AxA bypass and one (who had an aberrant right SCA) received a right CCA-right AxA-left CCA-left AxA bypass. A right AxA-left AxA bypass was performed in 14 out of the 16 patients with TEVAR landing in zone 2. Left SCA occlusion with a vascular plug was needed in 12 patients.

### Clinical outcomes

**Figure 3:**
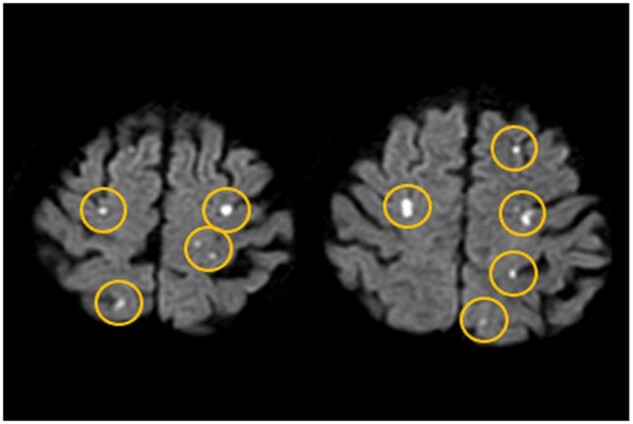
Impressive diffusion-weighted magnetic resonance imaging of multiple asymptomatic cerebral infarction after thoracic endovascular aortic repair.

All stent grafts were deployed successfully. DW-MRI confirmed new infarct lesions in 22 patients (34.3%), of whom 21 (32.8%) were asymptomatic and only one experienced (1.6%) symptomatic CI. This patient had ataxia 1 day after the procedure because of right cerebellar infarction and was fully recovered by rehabilitation. In patients who developed asymptomatic CI, DW-MRI revealed 44 new lesions in both the right and left hemispheres and in the anterior and posterior circulations (Fig. 3).

The prevalence of comorbidities was similar between the CI and non-CI groups. The grade of aortic arch atheroma was higher in the CI group than in the non-CI group (3.11 vs 2.63; *P* = 0.048). A significantly larger number of patients with proximal landing zones 0–2 were found in the CI group compared with the non-CI group (68.2% vs 11.9%; *P* < 0.001). As evidenced by DW-MRI, new lesions occurred in 4 of the 30 patients (13.3%) with TEVAR landing in zone 4 (*P* < 0.001), 15 of the 19 patients (78.9%) receiving TEVAR with debranching (*P* < 0.001) and 9 of the 12 cases (75.0%) with left SCA occlusion (*P* = 0.001).

**Figure 4:**
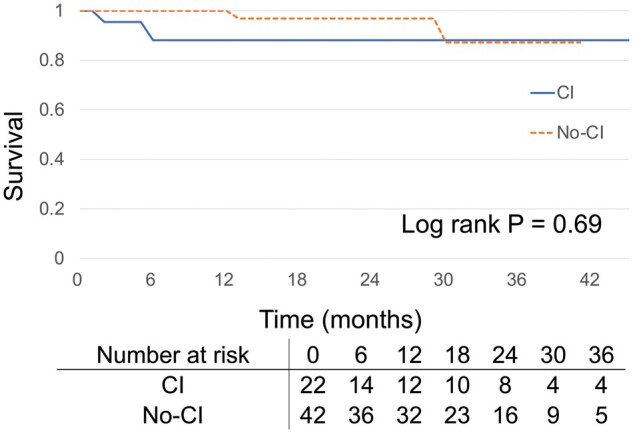
Kaplan–Meier all-cause survival curve of cerebral infarction versus no-cerebral infarction patients during follow-up (median 18.5 months, interquartile range 9.2–29.1 months).

There was one death (1.6%) within 30 days of surgery (Supplementary Material, Table S1), which resulted from sudden adrenal insufficiency after discharge. The patient had an asymptomatic, small, bilateral, parietal stroke, without any clinical symptoms or aorta-related events. The patient died 23 days after the procedure. Type I endoleak necessitated reintervention in 4 cases (6.3%), and 1 patient (1.6%) required open surgical hemi-arch replacement for retrograde type A dissection 9 months after the procedure. Kaplan–Meier survival analysis demonstrated no significant difference in all-cause survival between the 2 groups (*P* = 0.69; Fig. 4).

### Multivariate analysis

Multivariate analysis identified aortic arch debranching with an odds ratio (OR) of 16.7 and a 95% confidence interval of 3.11–89.3 (*P* = 0.001), left SCA occlusion (OR, 7.36; 95% CI, 1.28–42.3; *P* = 0.025) and atheroma grade ≥ 4 (OR, 5.63; 95% CI, 1.02–33.2; *P* = 0.048) as independent predictors of asymptomatic CI (Table 2).

**Table 2:** Multivariate analysis predictors for asymptomatic cerebral infarctions

Variable	OR	95% confidence interval	*P*-value
Debranching	16.7	3.11–89.3	0.001
Left SCA occlusion	7.36	1.28–42.3	0.025
Atheroma grade ≥4	5.63	1.02–33.2	0.048

OR: odds ratio; SCA: subclavian artery.

## DISCUSSION

The main finding of this study was that asymptomatic CI correlated with (i) TEVAR landing in zones 0–2, (ii) aortic arch debranching, (iii) left SCA occlusion with a vascular plug and (iv) high-grade aortic arch atheroma. Needless to say, debranching and left SCA occlusion are usually needed for the treatment of pathologies in zone 0–2. Briefly, both zone 0–2 endograft deployment and high-grade aortic arch atheroma were associated with the risk of developing CI. To the best of our knowledge, this is the largest-scale study in the literature.

A previous study reported the appearance of new foci on post-interventional cerebral DW-MRI in 63% (12 of 19) of patients [10]. Another report by Perera *et al.* [11] suggested a silent infarction rate of 68% (21 of 31) and a clinical stroke rate of 13% (4 of 31). In a study by Masada *et al.* [12], postoperative DW-MRI detected a total of 24 new lesions in 26% (5 of 19) of the patients undergoing elective debranching TEVAR. These studies were limited by the small number of patients enrolled.

Silent brain infarctions following endovascular repair of the aortic arch were recently reported by Charbonneau *et al.* [13] They found the incidence of silent CI was 50% (45 of 91), although all endografts were flushed with carbon dioxide before implantation. A study by Eleshra *et al.* [14] indicated that carbon dioxide flushing effectively exchanged trapped air for less harmful gas in endografts. In an experimental setting, several groups showed that the use of carbon dioxide for flushing significantly reduced the volume of gas released while opening the stent graft [15, 16]. This could be a promising approach to lower the risk of air embolism and stroke during TEVAR. In our study, we did not use carbon dioxide flushed devices, which might reduce the incidence of asymptomatic CI.

Asymptomatic CI has been reported to be associated with increased risks of future stroke, cognitive impairment and dementia in the long run [6, 7]. Previous studies have investigated asymptomatic CI following various catheter-based procedures. For example, cerebral DW-MRI has demonstrated new infarct lesions in 13–22% of patients after cardiac catheterization [2, 3], 68–93% of cases after transcatheter aortic valve replacement [4] and 33–49% of patients after carotid endarterectomy and carotid artery stenting [5].

Stroke after TEVAR is multifactorial. The proximal landing zone of stent grafts and left SCA coverage without revascularization have been reported as procedural risk factors for post-TEVAR cerebral embolization [17–20]. Another report suggested that occlusion of the supra-aortic trunks before stent-graft deployment reduces cerebral embolization [21].

Yoshitake *et al.* [22] described that debranching with TEVAR was associated with a high incidence of stroke. Concerning the design of the supra-aortic bypass for TEVAR landing in zone 2, subclavian-carotid transposition and carotid-subclavian bypass are widely used with documented long-term results [23]. However, we adopt AxA–AxA bypass instead of carotid-subclavian bypass because AxA–AxA bypass does not require touching left CCA, and the bypass can be established without disturbing cerebral perfusion during the procedure [24]. This extra-thoracic bypass in our series was extra-anatomical and relatively longer than the intrathoracic bypass. Our strategy of debranching and left SCA occlusion might affect the incidence of asymptomatic CI.

In this study, the presence of high-grade or mobile atheromas in the aortic arch was also associated with asymptomatic CI, which is consistent with previously reported results [25]. A possible explanation for this finding is that intraoperative instrumentation of the aorta might have disrupted aortic atheromas, making them became unstable or vulnerable to thromboembolism. A shaggy scoring system may be a useful method to predict CI following TEVAR [26].

Interestingly, our study also indicated that TEVAR landing in zone 4 posed a risk of asymptomatic CI. This finding could be attributed to the fact that TEVAR entails—irrespective of the intended landing zone—inserting a stiff guidewire across the aortic arch to help improve intraoperative stent-graft pushability, which may consequently lead to asymptomatic CI in the case of proximal landing zone 4. A previous study suggested there was no silent infarction in patients undergoing abdominal endovascular aortic repair, probably owing to the limited instrumentation of the aortic arch [11]. Therefore, guidewire manipulation across the aortic arch should be minimized to avoid CI in cases with TEVAR landing in zone 4.

Another unique finding of our study was that one of the patients treated with TEVAR after total arch replacement developed asymptomatic CI. In a study by Masada *et al.* [12], no new lesion was detected on cerebral DW-MRI in patients receiving TEVAR landing in zone 0 after replacement of the ascending aorta with a prosthetic graft. In a recent study, periprocedural cerebral embolic protection with the Claret Sentinel™ Device (Claret Medical Inc., CA, USA) proved to be of significant neurocognitive benefit to TEVAR patients [27]. This report suggested that valve tissues, myocardia and foreign materials are captured in the device as solid emboli. If these materials cause emboli, CI may develop even in cases with prior prosthetic graft replacement of aortic arch.

### Limitations

One of the limitations of the present study had to do with the small number of patients, which could adversely affect the robustness of our statistical analyses. The confidence intervals seem extremely wide; therefore, *P*-values may not be interpreted as confirmatory but descriptive. Other limitations of this study include debranching procedures, the strategy of device flushing and the timing of left SCA occlusion. We did not perform any analysis to differentiate between gaseous and atherothrombotic emboli on MRI. Our study was further limited by the lack of both long-term follow-up and detailed assessment of neurocognitive function. Therefore, the findings of this study should be validated through further clinical investigations.

## CONCLUSIONS

This study demonstrated that asymptomatic CI occurs frequently after TEVAR. Moreover, TEVAR landing in zone 0–2 and high-grade aortic arch atheroma were found to be significantly associated with asymptomatic CI.

## SUPPLEMENTARY MATERIAL

Supplementary material is available at *ICVTS* online.

**Conflict of** **interest:** none declared.

## Author contributions

**Sohsyu Kotani:** Conceptualization; Data curation; Formal analysis; Investigation; Methodology; Validation; Writing—original draft; Writing—review & editing. **Yoshito Inoue:** Project administration; Supervision; Writing—review & editing. **Naohiko Oki:** Investigation. **Hideki Yashiro:** Project administration. **Takashi Hachiya:** Project administration; Supervision.

## Reviewer information

Interactive CardioVascular and Thoracic Surgery thanks Luca Bertoglio, Mario Giovanni Gerardo D'Oria and the other anonymous reviewers for their contribution to the peer review process of this article.

## Supplementary Material

ivab240_Supplementary_Data

## Notes

The original draft of this manuscript was accepted as Plenary Citation at the AATS Aortic Symposium 2020, held at the New York Marriott Marquis Hotel on 23 and 24 April 2020.

This study was approved by the institutional review board of Hiratsuka City Hospital.

## ABBREVIATIONS

AxA: Axillary artery
CCA: Common carotid artery
CI: Cerebral infarction
DW-MRI: Diffusion-weighted magnetic resonance imaging
SCA: Subclavian artery
TEVAR: Thoracic endovascular aortic repair
