# Supplementary material for: Actual incidence of cerebral infarction after thoracic endovascular aortic repair: a magnetic resonance imaging study
Source: Interact Cardiovasc Thorac Surg. 2021 Oct 11;34(2):267–73. doi: 10.1093/icvts/ivab240 (PMC8766213; doi:10.1093/icvts/ivab240)
Supplement: ivab240_Supplementary_Data [file ivab240_supplementary_data.docx]

| Table S1. Clinical Outcome | |
| --- | --- |
|  | Overall N=64 |
| Technical success | 64 (100) |
|  |  |
| 30-day mortality | 1 (1.6) |
| In-hospital mortality | 0 (0) |
|  |  |
| Symptomatic CI | 1 (1.6) |
| Asymptomatic CI | 22 (34.4) |
| SCI | 0 (0) |
|  |  |
| Reintervention |  |
| Endoleak | 4 (6.3) |
| RTAD | 1 (1.6) |
| Open conversion | 0 (0) |

Values are n (%). CI, cerebral infarction; SCI, spinal cord injury; RTAD, r**etrograde** type A aortic dissection.
